# Evidence-Based Exercise Interventions and Fitness Technologies for Older Adults With Intact or Impaired Cognition

**DOI:** 10.1093/geroni/igaa057.3115

**Published:** 2020-12-16

**Authors:** Patricia Heyn, Stacy Andersen, Walter Boot

## Abstract

Effective exercise training and regular physical activity (PA) practice are important for healthy aging and are key modalities for chronic disease management. Approaches that enhance exercise prescriptions and adherence for older adults should be innovative and based on evidence. Thus, the exercise prescription should be tailored accordingly to the person’s health status, functional capacity, and living arrangements. To properly achieve the desirable health outcomes, the PA should be performed at higher intensity, greater frequency, and longer duration. National PA Guidelines recommend at least 150 minutes of moderate-intensity aerobic activity or 75 minutes of vigorous-intensity aerobic activity and at least two days of muscle-strengthening activities per week. Each prescribed modality (aerobic exercise, strength training, flexibility/stretching exercises, and balance training) has unique benefits. Therefore, it is critical to investigate and integrate novel techniques and tools to ensure adherence to the recommended PA guidelines. One of the presentations in this symposium, will highlight the research that has been evaluating for the last 15 years the effectiveness of the exercise randomized trial for older adults with/out cognitive impairments. The second presentation will introduce a novel technology paradigm designed to improve guidelines for exercise training as well as our understanding of how technology may motivate exercise behavior for older adults. The last presentation will discuss the design and methodological flaws that impact the outcomes of the exercise intervention by addressing the interpretations of non-findings and key factors affecting the desirable outcomes.

